# South African speech-language therapists’ views on dysphagia in head and neck cancer patients

**DOI:** 10.4102/sajcd.v72i2.1118

**Published:** 2025-11-24

**Authors:** Mishta Gungadeen, Jeannie van der Linde, Marien A. Graham, Bhavani S. Pillay

**Affiliations:** 1 Department of Speech-Language Pathology and Audiology, Faculty of Humanities, University of Pretoria, Pretoria, South Africa; 2 Department of Mathematics Education, College of Education, University of South Africa, Pretoria, South Africa

**Keywords:** speech-language therapist, head and neck cancer, dysphagia, swallowing management, survey

## Abstract

**Background:**

A significant gap in literature exists regarding speech-language therapists’ (SLTs’) guidelines for swallowing management in patients with head and neck cancer (HNC), particularly in low- and middle-income countries like South Africa.

**Objectives:**

This study explored a sample of South African SLTs’ views of swallowing management in patients with HNC.

**Method:**

A custom-designed online questionnaire was shared on social media platforms and completed by 14 South African SLTs with experience in swallowing management of patients with HNC. Quantitative data were analysed using descriptive statistics and structured tabular thematic analysis for qualitative data.

**Results:**

Participants (*n* = 14) believed they have a key role in the swallowing management of patients with HNC. Yet, they often face obstacles, such as late inclusion as a team member, cultural and linguistic barriers and/or limited access to resources and standard operating protocols. Most SLTs in this sample (*n* = 8) include instrumental assessments during initial evaluation. Treatment varied for each patient (e.g., type of surgical procedure [*n* = 14], anatomical and physiological changes [*n* = 14]). Speech-language therapists highlighted the importance of multidisciplinary collaboration, cultural and linguistic considerations and professional development.

**Conclusion:**

This population requires increased SLT involvement in multidisciplinary teams to ensure timely and appropriate evidence-based swallowing intervention.

**Contribution:**

This study provides a description of SLTs’ views on swallowing management of patients with HNC. Continued professional development is needed for South African SLTs to enhance evidence-based decision-making in HNC management, thus enhancing quality of care.

## Introduction

Head and neck cancer (HNC), including malignancies of the oral cavity, pharynx, larynx and nasopharynx, presents a unique set of challenges in medical and rehabilitative care because of its intricate anatomical and functional implications (Denaro et al., 2013; Divya & Sreya, 2020). Given the delicate interplay between HNC intervention and its sequelae, a comprehensive approach to post-operative care is crucial. Speech-language therapists (SLTs) are pivotal in managing swallowing disorders and enhancing patient outcomes (Zuydam et al., 2022).

Surgical resection of tumours, a common HNC intervention, can significantly disrupt swallow coordination, which requires precise muscle action and effective sensory feedback (Baijens et al., 2021). Beyond surgery, radiation therapy often causes collateral damage to surrounding tissues. This can lead to radiation-induced mucositis, fibrosis, xerostomia, swelling and skin reactions, further compromising swallowing function (Baijens et al., 2021; Jaison Varghese et al., 2024). Specifically, radio-induced tissue fibrosis may negatively alter irradiated muscles, resulting in slower contraction speeds, reduced swallowing movement and delayed pharyngeal responses, thereby increasing a patient’s risk of aspiration (Beuren et al., 2023).

A common structural impairment following a laryngectomy is pharyngocutaneous fistulae (PCF), a complication in the pharyngeal mucosal lining (Aires et al., 2015; Singh et al., 2021). For many years, delayed oral eating (7–14 days) post-laryngectomy has been standard practice to reduce the risk of PCF formation (Singh et al., 2021). Despite this, recent research suggests that early oral feeding may reduce PCF development (Benali et al., 2022; Sousa et al., 2015). This is because the presence of food or water can dilute saliva, reducing its pH level and action at the suture line (Aires et al., 2015; Singh et al., 2021; Sousa et al., 2015). Early oral feeding also offers several advantages, including shorter hospital stays, reduced psychological stress and hospital costs and potentially earlier restoration of swallowing function (Sousa et al., 2015). However, delayed oral intake is often still preferred because of a lack of conclusive evidence supporting early feeding (Benali et al., 2022).

During periods of delayed oral feeding, a nasogastric tube (NGT) is commonly inserted to ensure sufficient nutrition (Suslu & Hoşal, 2016). Unfortunately, an NGT can increase trauma to the pharyngeal suture line over time or damage the pharyngeal mucosa because of gastroesophageal reflux, potentially contributing to fistula formation (Suslu & Hoşal, 2016). When long-term nutritional support (30 days or more) is required, a percutaneous endoscopic gastrostomy (PEG) is the preferred option (Yanni et al., 2019).

There are conflicting perspectives regarding the optimal time to introduce oral nutrition. Although Soria et al. (2017) support enteral nutrition, Beuren et al. (2023) assert that prioritising oral nutrition and incorporating prophylactic swallowing exercises throughout intervention can improve a patient’s quality of life, swallowing function, diet consistency and reduce reliance on alternative nutrition. Additionally, reduced use of oral muscles may lead to early atrophy, manifesting as poor oral-motor control, low strength and extreme fatigue (Kristensen et al., 2020). Therefore, a team decision is vital to ensure that each patient receives tailored intervention.

Swallowing management is a multidisciplinary team effort, often including an oncologist, maxillofacial surgeon, otolaryngologist, dietitian, physiotherapist and SLT (Zuydam et al., 2022). Intervention ideally begins before the surgical procedure, during which a baseline of swallowing abilities is obtained. Literature outlines rehabilitative techniques for swallowing that include compensatory strategies, therapeutic exercises, diet modifications and patient education (Benali et al., 2022; Clarke et al., 2016). The minimal inclusion of SLTs in some settings can prevent a complete intervention plan for this population, as swallowing management should begin pre-operatively and be adjusted as necessary thereafter (Galbiatti et al., 2013; Zuydam et al., 2022).

South Africa, considered an upper-middle-income country with low-resource settings, faces a challenged healthcare system that leads to limited access to allied healthcare services and untimely oncological management (Pramesh et al., 2022; Seedat et al., 2023). Given that the incidence of HNC makes it the third most common cancer in men and sixth most common in women globally, low- and middle-income countries must have literature for informed decision-making in post-operative swallowing management (Sharma et al., 2020). Research targeting context-specific challenges and effective treatment techniques for HNC in low- and middle-income settings aims to improve intervention for this population (Sharma et al., 2020).

This study aimed to describe SLTs’ views of swallowing management of the HNC population in South Africa.

## Research methods and design

### Study design and setting

A descriptive survey research design was employed. A questionnaire was utilised as the data-collection instrument, allowing the researcher to obtain anonymous data (Kumar, 2013). The same constructs were explored using both open- and closed-ended questions, thus allowing for rich data triangulation (Brink et al., 2018). Distributing the questionnaire electronically was an inexpensive data-collection method and aligned with contemporary communication trends (Brink et al., 2018).

### Study population and sampling strategy

A non-probability, purposive sampling method was employed to select participants for this study, as it enabled the selection of a sample population (i.e. SLTs) who are more exposed to, and knowledgeable in, the swallowing management practices of patients with HNC (Brink et al., 2018). Participants were required to be qualified SLTs in South Africa, with a minimum of 6 months of clinical experience in the swallowing management of patients with HNC who have undergone total laryngectomy, partial laryngectomy, total pharyngectomy, partial pharyngectomy and/or pharyngolaryngectomy. Speech-language therapists should have worked with patients who had HNC with swallowing difficulties in the last 3–5 years and have access to an electronic device, as the questionnaire was only available online. As managing the speech and swallowing complications of this population is a specialised area for SLTs, a small sample for this study was expected (Logan & Landera, 2021).

### Survey

A custom-designed electronic questionnaire was used to gather data from the target population. The 36-item questionnaire included four sections based on the published work of Denaro et al. (2013), Roe et al. (2012), Clarke et al. (2016), Krisciunas et al. (2019), Loewen et al. (2021) and Benali et al. (2022). There were 32 closed-ended questions and four open-ended questions included. Sections consisted of participants’ demographic information; identifying and referring HNC patients suitable for swallowing management and assessment and treatment approaches for patients with HNC. A pre-testing of the questionnaire took place by requesting two SLTs with postgraduate degrees in Speech-Language Pathology, and 2–3 years of experience in the swallowing management of patients with HNC, to determine whether the clarity of the questions, layout and ease of completion were appropriate for the target population. Feedback on the provisional questionnaire was obtained (Brink et al., 2018). The sections were reduced from five to four to ensure more concise questioning and ease of completion. Seven ‘yes’ or ‘no’ questions were part of the survey, and 10 multiple-choice questions where more than one option selection was allowed. Additionally, two Likert scales and two matrix questions were included.

### Data collection

An infographic, including the survey link, was circulated on various social media platforms, professional networks and professional associations, with two questions embedded to determine eligibility. The questions were: ‘Are you a qualified SLT?’ and ‘Do you have a minimum of six months experience working with HNC patients?’ Informed consent was embedded in the online questionnaire. The online medium (via Qualtrics XM software) enabled a larger population of SLTs to be reached (Brink et al., 2018).

### Data analysis

Data were analysed using descriptive statistics, including frequency distributions, which represent the number of times participants chose a response (Brink et al., 2018). The data were captured on Microsoft Excel spreadsheets and analysed using the IBM Statistical Package for Social Sciences (SPSS) programme in collaboration with a statistician. Open-ended questions were analysed qualitatively, using structured tabular thematic analysis that assisted the researcher in identifying any patterns present in the qualitative data (Robinson, 2022).

### Ethical considerations

The study obtained Institutional Review Board (IRB) clearance from a large north-eastern public university in South Africa 30 April 2024 (reference number: HUM004/1223).

Adhering to the *Protection of Personal Information Act* (POPIA, 2019), participants’ personal information was not used, thus safeguarding their identities. To ensure this, each participant received an alphanumeric code, for example A001, that was used throughout data management and reporting of results. The university’s research data management policy requires research data to be stored for a minimum of 10 years upon completion of the study. During this period, data will be kept electronically as stipulated by the University’s Institutional Research Data Management System and accessible via the University’s Research Data Management Repository.

## Results

An initial description of the South African SLT sample is provided. Subsequently, results are presented through the lens of a continuum of care in swallowing intervention, covering referral pathways, assessment and treatment. Finally, the specific role of the SLT and their professional development opportunities is detailed.

### Demographics

A total of 14 responses were received and statistically analysed. Participants were aged between 22 and 49 years. Half of the participants (*n* = 7) served the HNC population for less than 5 years and the remaining half (*n* = 7) for more than 5 years (Table 1). Work settings, where respondents could choose more than one setting, were mainly the private sector (*n* = 8), followed closely by the public sector (*n* = 7). However, because of the multi-response option provided, it was noted that one participant reported having worked in public health for 17 years and recently joined the private sector in the last 2 years. Furthermore, other participants (*n* = 2) indicated more than one place of work, with one participant working in private and public health, and the other working in the former as well as a tertiary institution.

**TABLE 1:** Participant demographics (*N* = 14).

Variables	*n*
**Age (years)**	14
22–29	2
30–39	9
40–49	3
**Highest qualification**	14
Bachelor’s degree	8
Post-graduate degree	6
**Years practising as an SLT**	14
< 5	1
> 5	13
**Years managing patients with HNC**	14
< 5	7
5–10	3
10–15	1
> 15	3
**Province**	14
Gauteng	6
KwaZulu-Natal	4
Western Cape	3
Eastern Cape	1
**Number of patients with HNC seen per year**	14
0–20	6
20–40	5
50+	3
**Place of work** †	16
Private sector	8
Public sector	7
Tertiary institution	1

SLT, speech-language therapists; HNC, head and neck cancer.

† , Biographical questions where more than one response was allowed, which could reflect a total of more than *n* = 14 and percentages adding up to more than 100%.

### Referral pathways

Half of the participants used screening measures to identify patients with HNC who qualify for post-operative swallowing management (Table 2). The most common screening measures were bedside swallow evaluations (*n* = 2), Eating Assessment Tool (EAT-10) (*n* = 2), Mann Assessment of Swallowing Ability – Cancer (MASA-C) (*n* = 2) and multidisciplinary team discussions (*n* = 2). All participants (*n* = 14) received referrals from other healthcare professionals, particularly the oncologist (*n* = 12), ear, nose, and throat specialist (ENT) (*n* = 12) and physiotherapist (*n* = 9). Six participants reported receiving referrals routinely for pre-surgical intervention, whereas another six received them only when swallowing difficulties were present. One participant reported receiving the referral for post-surgical intervention only.

**TABLE 2:** Referral pathways (*N* = 14).

Referral pathways	*n*
**Part of a healthcare team involved in providing care and intervention to patients with HNC**	14
Always	6
Often	6
Sometimes	2
**Use of screening measures to identify patients with HNC requiring swallowing intervention**	14
Yes	7
No	7
**Referrals received from other healthcare professionals for patients with HNC**	14
Yes	7
**Healthcare professionals’ referrals are received** †	66
Oncologist	12
Otolaryngologist	19
Dietitian	10
Physiotherapist	9
Maxillofacial surgeon	5
Occupational therapist	5
Plastic surgeon	4
Radiologist	1
Social worker, nurse, hospice, home-based carer	1
**Time at which referral is typically received**	14
Pre-surgical intervention	6
Only when swallowing difficulties are apparent	6
Post-surgical intervention	2

HNC, head and neck cancer.

† , Participants could select more than one option, which could reflect a total of more than *n* = 14 and percentages adding up to more than 100%.

### Assessment

Speech-language therapists from this study reported that their initial dysphagia assessment (Table 3) typically comprises: a case history interview (*n* = 14), patient orientation (*n* = 13), medical stability for dysphagia assessment (*n* = 14), oral-motor examination (*n* = 14), bedside swallowing evaluation (*n* = 14) and instrumental swallowing assessment (*n* = 8). Three participants elaborated that they would also review the patient’s x-rays and conduct an instrumental swallow assessment only 4 days post-operatively, at the earliest.

**TABLE 3:** Assessment of dysphagia in patients with head and neck cancer (*N* = 14).

Assessment factors	*n*
**Components of initial assessment**?	80
Case history	14
Medically stable for dysphagia assessment	14
Oral-motor examination	14
Bedside swallowing evaluation	14
Patient orientation	13
Instrumental swallowing assessment	8
X-rays	3
**Use of instrumental swallowing assessments**	14
No	11
Yes	3
**Factors influencing decision-making on instrumental swallowing assessments**?	113
Abnormal vocal quality after swallowing (i.e., wet or gurgling voice) and/or dysphonia	14
Querying silent aspiration	14
Coughing, choking or throat clearing before, during or after, swallowing	13
Inability to control and/or coordinate food, liquids or saliva in the oral cavity	11
Complaint of food ‘sticking’ in the throat	10
Complaint of difficulty swallowing	9
Nasal regurgitation	9
Weight loss	8
Prolonged enteral feeds	8
Build-up or congestion after a meal	6
Drooling	5
Pocketing of food in cheek	2
Oxygen saturation levels	2
Excessive chewing	1
Presence of a tracheostomy	1
**Tools used during initial assessment**?	65
***Swallowing***	
100 mL water swallow test and/or timed water swallow test (TWST)	10
Mann Assessment of Swallowing Ability (MASA) and/or Mann Assessment of Swallowing Ability – Cancer (MASA-C)	7
Functional Intraoral Glasgow Scale (FIGS)	1
Cranial nerve exam	1
Performance Status Scale for Head and Neck Cancer (PSS-HN)	1
***Voice***	
Spontaneous speech sample and/or rainbow passage	12
Auditory-perceptual assessment for voice and resonance	9
Instrumental voice assessment(s)	3
GRBASI 4-point rating scale	1
***Cognition***	
Montreal Cognitive Assessment (MoCA) and/or Mini-Mental Status Examination (MMSE)	6
***Quality of life via patient-reported measures***	
Eating Assessment Tool (EAT-10)	4
Area not currently included in assessment protocol	4
Use of subjective patient remarks	2
The Swallowing Quality-of-Life Questionnaire	1
Rosenbek Penetration-Aspiration scale	1
MD Anderson Dysphagia Inventory (MDADI)	1
Functional Assessment of Cancer Therapy – Head and Neck (FACT-HN)	1

MD, Monroe Dunaway.

?, Participants could select more than one option, which could reflect a total of more than *n* = 14 and percentages adding up to more than 100%.

Interestingly, majority of participants (*n* = 11) would not conduct an instrumental swallowing assessment on all their patients during the initial stages of intervention, and the latter (*n* = 3) reported that they would. The pertinent clinical factors influencing their decision-making included querying silent aspiration (*n* = 14); coughing, choking or throat clearing before, during or after swallowing (*n* = 13); abnormal vocal quality after swallowing (i.e. wet or gurgling voice) and/or dysphonia (*n* = 14); inability to control and/or coordinate food, liquids or saliva in the oral cavity (*n* = 11); complaint of food ‘sticking’ in the throat (*n* = 10); complaint of difficulty swallowing (*n* = 9); nasal regurgitation (*n* = 9); weight loss (*n* = 8) and prolonged enteral feeds (*n* = 8). The assessment tools used are summarised in Table 3.

### Treatment

Some of the most common surgical procedures SLTs reportedly seen in patients with HNC are total laryngectomy (*n* = 12), partial laryngectomy (*n* = 8), total pharyngectomy (*n* = 9), partial pharyngectomy (*n* = 9) and pharyngolaryngectomy (*n* = 9). All respondents(*n* = 14) agreed that their swallowing intervention would differ according to the surgical procedure.

#### Therapeutic interventions

The reported aspects of treatment approaches used when managing HNC patients with swallowing impairments included a range of techniques: compensatory strategies (*n* = 14), swallow manoeuvres (*n* = 14), non-swallow exercises (*n* = 14), stretches (*n* = 5) and patient education (*n* = 3).

#### Individualised swallowing management approaches

Speech-language therapists emphasised that swallowing management for patients with HNC is highly individualised, adapting to the unique anatomical and physiological changes resulting from each surgical procedure. As one respondent commented:

‘Each surgical procedure results in different anatomical and physiological changes. The approach, the degree of the removal and even the knock-on effects of previous procedures can impact a “routine” approach. You have to treat according to the deficits your assessment picks up, use objective measures to guide your plan and work based on facts.’ (Respondent 13, SLT, treating patients with HNC for 1 year)

This personalised approach extends to the nature of the surgery itself, as therapy is often contingent on the specific difficulties identified during instrumental assessments, such as a modified barium swallow examination, and the need for further treatments like radiation or chemotherapy:

‘My swallowing therapy is determined by the nature of the surgery and the difficulties shown on the modified barium swallow examination, and also whether or not further treatment, e.g., radiation or chemotherapy are required.’ (Respondent 11, SLT, treating patients with HNC for 6 years)

The majority of participants (Table 4) reported not having a workplace-stipulated swallowing management protocol, which was viewed as somewhat advantageous, allowing for a unique, contextualised approach:

‘… each patient is treated uniquely. I think the other aspect is that this is a fairly new field for our country and surgeons. As their protocols develop, they guide us and we can make educated protocols together.’ (Respondent 13, SLT, treating patients with HNC for 1 year)

**TABLE 4:** Treatment of dysphagia in patients with head and neck cancer (*N* = 14).

Treatment	*n*
**Aspects included in treatment approach when managing patients with HNC who present with dysphagia** †	48
Compensatory techniques (e.g., position changes, bolus consistency changes, liquid wash)	14
Swallow manoeuvres (e.g., Mendelsohn, effortful swallow, super supraglottic)	14
Non-swallow exercise (e.g., tongue base exercises, laryngeal or pharyngeal exercises, myofascial therapy)	12
Stretches (e.g., neck, jaw, tongue)	5
Patient education	3
**Duration recommended that patients with HNC continue with swallowing exercises**	14
Continuous as a maintenance programme	8
Until swallowing difficulty resolves	5
6 months post-treatment	1
**Involvement of SLTs in pre- and post-counselling of PEG-tube placement**	14
Often	6
Sometimes	4
Always	3
Rarely	1
**Recommendations for patients with HNC using enteral feeds during treatment and/or post-treatment**	14
Only use the enteral feed when absolutely necessary	10
Use the enteral feed conservatively	2
Use the enteral feed as much as possible	2
**Recommended guidelines for enteral feeds in patients with HNC who receive radiation therapy**	14
All patients are recommended to receive enteral feeds only when needed	9
Some patients are recommended to receive enteral feeds prophylactically according to specific institutional guidelines.	3
All patients are recommended to receive enteral feeds prophylactically (before or during the first week of radiation therapy).	2
**Variation in swallowing intervention according to the surgical procedure**	14
Yes	14

HNC, head and neck cancer, SLT, speech-language therapists; PEG, percutaneous endoscopic gastrostomy.

† , Participants could select more than one option, which could reflect a total of more than *n* = 14 and percentages adding up to more than 100%.

Some guidelines were also viewed as challenging as a participant shared that:

‘… one protocol that is a hard rule for laryngectomy patients, is that they are not to eat or undergo videoswallow before 14 days postoperatively.’ (Respondent 13, SLT, treating patients with HNC for 1 year)

#### Enteral nutrition

When HNC patients are utilising enteral feeds during, or post-treatment, five respondents recommended that this method only be used when absolutely necessary. Other participants supported this cautious approach, emphasising the importance of maintaining oral muscle function:

‘PEG [*percutaneous endoscopic gastrostomy*] tubes are sometimes placed prophylactically. Patients are encouraged to keep swallow muscles as active as possible but to supplement with PEG tube feeding’ (Respondent 2, SLT, treating patients with HNC for 6 years).

Effective multidisciplinary communication was also highlighted as crucial for optimal patient management:

‘Communication between doctors, dietician and SLT is essential to ensure optimising caloric intake as well as swallow function’ (Respondent 2, SLT, treating patients with HNC for 6 years).

These recommendations align with the participants’ views for enteral feeds in patients with HNC receiving radiation therapy – only when needed, as agreed upon by nine participants.

#### Pre-operative counselling

When participants were asked how their intervention would differ if a referral was received during the pre-operative stage, the qualitative data revealed a common theme: patient counselling. This proactive approach allows for greater patient involvement and consideration of pre-existing difficulties:

‘More extensive pre-operative counselling where the patient can actively be involved in the decision making and planning process for rehabilitation, allows for closer consideration of pre-operative difficulties in relation to post-operative difficulties.’ (Respondent 5, SLT, treating patients with HNC for 3 years)

Another respondent highlighted the dual nature of this early counselling:

‘Pre-operative treatment mainly entails counselling on current abilities and possible changes post-operatively. My role is also in counselling the patient on their voice and/or communication options post-operatively in aiding to make an informed decision [*especially in total laryngectomy*].’ (Respondent 10, SLT, treating patients with HNC for 2 years)

#### Cultural and linguistic considerations

After careful analysis of the qualitative data regarding cultural and linguistic considerations for HNC patients, two common themes emerged: (1) holistic management that ensures contextually appropriate access to services and dietary modifications and (2) the language of patient education. Given South Africa’s diverse cultural and linguistic landscape, tailoring interventions to be contextually appropriate are crucial when treating HNC patients.

This is exemplified by one respondent’s view on holistic management:

‘Holistic management that is contextually appropriate [*is important*] considering the patient’s financial means, home setting, ability to access running water and electricity, who is available to help at home and/or caregivers, patient’s ability to frequently follow up for speech therapy, the patient’s literacy level, and whether they need written or visual instruction and/or handouts.’ (Respondent 10, SLT, treating patients with HNC for 2 years)

#### Role of the speech-language therapist

The perceived key responsibilities of SLTs included screening patients with HNC for swallowing intervention (*n* = 14), conducting swallowing assessments collaboratively within the healthcare team (*n* = 14), diagnosing swallowing abilities – which all participants (*n* = 14) agreed were extremely important. Providing referrals for further interventions when necessary varied between extremely important (*n* = 11) and very important (*n* = 3). When determining the need for instrumental swallowing assessments, most participants (*n* = 12 out of 14) noted their role to be extremely important, with the latter selected very (*n* = 1) and moderately important (*n* = 1). Speech-language therapists valued consulting during family meetings about enteral feeding options as extremely important (*n* = 10) and very important (*n* = 4). Prophylactic swallowing exercises and counselling on safe oral intake and aspiration prevention were also highlighted as crucial aspects of patient care. The former, being prophylactic swallowing exercises, were rated as extremely (*n* = 7), very (*n* = 5) and moderately (*n* = 2) important and the latter, being counselling on safe oral intake and aspiration prevention, as ‘extremely’ (*n* = 13) and ‘very important’ (*n* = 1).

#### Professional development

**FIGURE 1:**
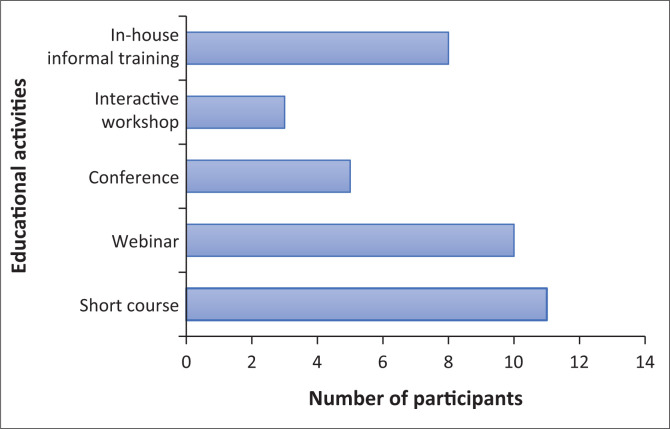
Types of educational activities attended.

Most (*n* = 12) respondents who participated in this study had attended a specialised professional education activity regarding HNC management (Figure 1).

**FIGURE 2:**
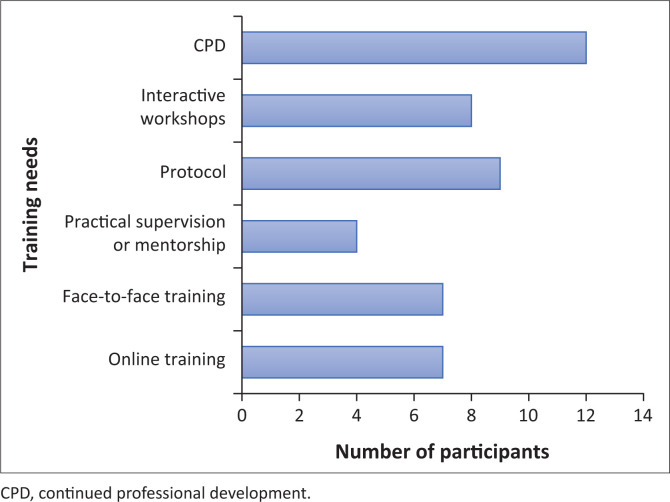
Types of educational training needs.

All participants (*n* = 14) agreed that they wanted to know more about the swallowing management of patients with HNC and reported on their method of receiving this education, as seen in Figure 2.

All participants (*n* = 14) acknowledged the importance of being knowledgeable about providing swallowing rehabilitation to patients with HNC in their current healthcare. Furthermore, SLTs rated having sufficient experience and feeling confident in providing swallowing rehabilitation in their current healthcare setting as ‘extremely’ (*n* = 11) and ‘very important’ (*n* = 3).

Despite this recognised importance, when SLTs were asked about gaps in their knowledge, experience or clinical skills in providing swallowing management to patients with HNC, two themes emerged: *Current trends in swallowing management of patients with HNC* and *variations in swallowing impairments according to the surgical procedure*. These concerns are reflected in participants’ responses highlighted further in the text, where the first quote refers to the former theme and the second quote the latter:

‘Expiratory Muscle training and/or TMJ management within SA Context and/or Humidification management and/or Trachea management.’ (Respondent 8, SLT, practising for 6 years and seeing patients with HNC for past 3 years)

‘To which extent swallowing function changes or fluctuates during radiation therapy, as well as which manoeuvres or compensatory strategies are proven best or standard for which operations and which are contra-indicated.’ (Respondent 10, SLT, practising for 3 years and seeing patients with HNC for past 2 years)

## Discussion

This study aimed to explore the practices and perspectives of SLTs in managing swallowing difficulties in patients with HNC in South Africa. The results were varying, thus offering a broad perspective on current practices in HNC swallowing management.

Data from this study revealed that the perceived role of the South African SLT included the pre-rehabilitative stage and consisted of assessing, diagnosing and treating swallowing and voice impairments in patients with HNC. Furthermore, SLTs also reported that they play a vital role in providing appropriate ongoing and alternative feeding options within their area of expertise and scope of practice. Interestingly, when comparing a study by Logan and Landera (2021), it was found that less than half of the SLTs who participated in their study completed prerehabilitative evaluations. According to the National Comprehensive Cancer Network based in the United States of America, a pretreatment evaluation of speech and swallowing function in this patient population is recommended to obtain a baseline (National Comprehensive Cancer Network, 2025). This is consistent with literature that supports the inclusion of the SLT in the prerehabilitative stages of treatment as it impacts clinical decision-making and simplifies patient care, thereby ensuring evidence-based care (Starmer et al., 2011).

The referral pathways outlined in the current study for swallowing management in the HNC population were notably collaborative, proving advantageous to the multidisciplinary team effort required for the intervention of patients with HNC and consistent with ensuring the highest quality of care (Zuydam et al., 2022). Interestingly, there was no clear consensus on when referrals were typically received. This variability may reflect differences in institutional protocols and the recognition of dysphagia as an ongoing condition in the HNC treatment continuum (Benali et al., 2022). Such variability underscores the necessity for improved interprofessional communication and standardised referral guidelines.

To align with the World Health Organization’s (WHO) International Classification of Functioning, Disability and Health (ICF) framework, SLTs assessed (initial and reassessment) patients with HNC by examining body structures and functions through oral-facial musculature for swallowing and voice. Activities were investigated via oral intake limitations using clinical swallowing evaluations and self-reports. Aspects of participation through patient-reported measures surrounding restrictions in shared social experiences, like family meals and dining out. Speech and language therapists managing patients with HNC reported using various assessment tools. Despite this variation, the areas evaluated remained constant: swallowing, voice, cognition and quality of life via patient-reported measures. The ICF framework allows for consistency to be maintained, ensuring that patients with HNC are viewed through a holistic lens by not only considering the anatomical and physiological components of swallowing along with the dynamic nature of surgical and radiological interventions, but also the psychological and social impact on their lives (Tschiesner et al., 2010).

As seen in the results, most participants did not always conduct an instrumental swallowing assessment on their patients with HNC. Research shows that an instrumental swallow assessment allows clinicians to understand the pathophysiology of the HNC population, improve oral intake, decrease the risk of silent aspiration and establish rehabilitation protocols (Vieira et al., 2024). It is more prominently used when querying dysfunction of the upper oesophageal sphincter because of oropharyngeal dysphagia in this target population, as such dysfunction is multifactorial with HNC (Baijens et al., 2021).

Beyond a patient’s clinical presentation, the sample of SLTs reported that limited access to instrumental swallowing evaluations and efforts to reduce radiation exposure influenced their decision not to conduct these assessments. However, the knowledge gaps that participants expressed, particularly concerning surgery-specific swallowing rehabilitation and current trends in HNC swallowing management, suggest that the potential benefits of instrumental swallow assessments may not be fully recognised. The treatment strategies for managing dysphagia in HNC patients were tailored to the individual’s surgical procedure and the anatomical changes, thereafter; as reported by all participants. Common procedures such as total laryngectomy, partial laryngectomy and pharyngectomy necessitated different swallowing interventions based on the anatomical changes resulting from surgery. Patterson and Lawton (2023) support a baseline measure of swallowing and psychosocial functioning, tumour site details and the type of radiotherapy allows for individualised swallowing management in patients with HNC. These authors elaborate on how a tailored treatment approach may assist in reducing mental and exercise burden, as intensive loading does not apply to all patients (Patterson & Lawton, 2023). It is vital to note that there are no published protocols in South Africa regarding the swallowing and communication management in patients with HNC. Furthermore, there is a scarcity of internationally available resources that may be contextually appropriate for South Africa (Coutts et al., 2022) given resource constraints and service delivery barriers. For example, Clarke et al. (2016) published speech and swallowing guidelines for the HNC population within a multidisciplinary context in the United Kingdom. These guidelines highlighted the importance of obtaining a baseline of swallowing function prior to, and following oncology intervention; however, duration and dosage of treatment were not specified (Blyth et al., 2024).

A key component of treatment was pre-operative counselling, with all participants noting that providing patients with a clear understanding of the expected changes in swallowing function was critical for managing expectations and improving post-surgical outcomes. This component assists SLTs in obtaining a baseline measure of the patient’s skills pre-operatively to compare with post-operative abilities and allows for patient education on the areas of change (Beuren et al., 2023). Lack of information surrounding oncological intervention allows fear to arise in the patient and negatively impacts treatment compliance (Lourens, 2013).

While international research underscores the benefits of prehabilitation, routine screening and equitable access to SLT services (Amaral et al., 2022; Karasik et al., 2025), current South African trends remain largely reactive. Most patients in the HNC population present at advanced stages and receive limited rehabilitative support post-treatment (Coutts et al., 2023). Such limitations are typically because of poorly resourced public healthcare facilities, inconsistent documentation and geographical barriers (Coutts et al., 2023).

A proactive approach to patient education aligns with current trends in swallowing rehabilitation, where pre-surgical counselling is seen as a vital part of the rehabilitation process (Patterson & Lawton, 2023). It assists in establishing a baseline, preparing patients for the post-operative rehabilitation process, ensuring they are actively involved in the decision-making process, reducing their psychological strain and enhancing their prognosis (Lourens, 2013; Patterson & Lawton, 2023). Lourens (2013) found that oncology social workers play a collaborative role with SLTs and are an asset to the multidisciplinary team, as they help ensure patients with HNC and their families are well informed about the intervention journey, even in the patient’s traditional language when necessary.

Enteral nutrition is a common intervention in managing nutrition for patients undergoing treatment for HNC (Soria et al., 2017). However, there was consensus among SLTs that enteral feeding should be used conservatively and only when necessary. This recommendation reflects the belief that maintaining oral intake is essential to preserving swallowing function, improving quality of life and minimising the use of enteral nutrition (Beuren et al., 2023). Additionally, the reduced use of oral muscles may result in early atrophy, manifesting as poor oral-motor control, low strength and extreme fatigue (Kristensen et al., 2020). Some negative factors of enteral nutrition may include gastrointestinal tract complications such as abdominal distention and regurgitation and a poor psychosocial state because of social exclusion from receiving nutrition via an atypical method (Gliwska et al., 2023).

Despite the available scientific evidence advocating the efficacy of enteral nutrition in oncology intervention, clinicians need to consider its potential negative effects on an individual’s quality of life (Gliwska et al., 2023). Cultural and linguistic considerations emerged as a critical aspect of managing patients with HNC within the South African context. Respondents highlighted the importance of adopting a holistic, culturally sensitive approach to care. This is crucial because cultural beliefs can act as a barrier to healthcare access, influencing how a health condition is understood and the services patients seek (Lourens, 2013). Similar to disparities noted internationally – particularly among minority and underserved populations, South African patients face systemic inequities that compromise dysphagia outcomes (Coutts et al., 2022; Karasik et al., 2025). These findings emphasise the need for contextually relevant, healthcare pathways that prioritise early dysphagia surveillance and integrate the role of the SLT as a standard component of HNC care across all stages of treatment.

Participants detailed that recognising patients’ diverse socioeconomic realities, such as limited access to basic amenities, and adjusting dietary modifications to be contextually appropriate contribute to culturally sensitive healthcare. This is especially important, considering the National Health Insurance (NHI) Bill, which was passed in May 2024, aiming to provide equitable access to quality healthcare services. Njilo and Ross (2025) found that Zambia’s NHI scheme enhanced healthcare accessibility but was fraught with obstacles such as poor quality of care, inadequate involvement of stakeholders and insufficient funding. To ensure the success of speech-language therapy services in South Africa during the implementation of the NHI, the scope of practice for SLTs needs to be protected within the NHI framework. Professional bodies should be well involved in policy decisions, and more SLT training and employment opportunities should be made available to improve services in the overburdened public sector (Njilo & Ross, 2025).

Language barriers were also noted as a significant challenge, with SLTs emphasising the need for clear communication and the use of accessible educational materials with varying literacy levels. This is particularly important in a multicultural society like South Africa, where language and cultural practices significantly influence how patients perceive and engage with healthcare interventions (Seedat et al., 2023).

The majority of participants attended specialised education activities related to HNC, indicating a commitment to continued professional development. However, significant gaps in knowledge were recognised, particularly regarding current trends in swallowing rehabilitation and how swallowing impairments vary according to the surgical procedure. The desire for further education highlights the importance of continued professional development to ensure that SLTs remain informed about evidence-based practices and emerging developments in swallowing management of the HNC population (Bhattarai, 2022). Providing targeted education and training on these topics could enhance clinical confidence and improve the quality of care provided to patients with HNC (Patterson & Lawton, 2023).

### Strengths and limitations

This study was able to yield a response only from 14 South African SLTs. A sample of this size may be susceptible to bias, given that small sample sizes may reduce reliability, thus leading to increased variability. Despite obtaining a small sample size, it should be noted that the swallowing management of patients with HNC is a niche area and not all SLTs are part of this specialist field. Recall bias from personal experiences with patients with HNC may have occurred and influenced participants’ responses (Brink et al., 2018). To the researchers’ knowledge, this study was the first of its kind to be conducted in a low-to-middle-income country; therefore, adding value to the current body of literature. Awareness of the lack of guidelines for South African SLTs was highlighted, thus establishing the need for continuous professional development in this area.

## Conclusion

This study highlights the essential role of South African SLTs in managing dysphagia in patients with HNC, particularly in pre-rehabilitation assessment, treatment planning and patient education. While current practices partly align with international recommendations, inconsistencies in instrumental assessment use and referral timing reflect systemic limitations and resource constraints. Cultural, linguistic and socioeconomic factors further shape care delivery, emphasising the need for contextually responsive and culturally sensitive approaches. The identified gaps in specialist knowledge highlight the importance of continued professional development and access to tailored training. Standardised clinical pathways, improved multidisciplinary collaboration and increased access to diagnostic tools are crucial for advancing equitable, evidence-based care for patients with HNC in South Africa.
